# Resting State Brain Function Analysis Using Concurrent BOLD in ASL Perfusion fMRI

**DOI:** 10.1371/journal.pone.0065884

**Published:** 2013-06-04

**Authors:** Senhua Zhu, Zhuo Fang, Siyuan Hu, Ze Wang, Hengyi Rao

**Affiliations:** 1 Department of psychology, Sun Yat-Sen University, Guangzhou, Guangdong, China; 2 Center for functional Neuroimaging, Departments of Neurology, University of Pennsylvania Perelman School of Medicine, Philadelphia, Pennsylvania, United States of America; 3 Department of Psychiatry, University of Pennsylvania Perelman School of Medicine, Philadelphia, Pennsylvania, United States of America; 4 State Key Laboratory of Cognitive Neuroscience and Learning, Beijing Normal University, Beijing, China; University of Massachusetts Medical School, United States of America

## Abstract

The past decade has seen astounding discoveries about resting-state brain activity patterns in normal brain as well as their alterations in brain diseases. While the vast majority of resting-state studies are based on the blood-oxygen-level-dependent (BOLD) functional MRI (fMRI), arterial spin labeling (ASL) perfusion fMRI can simultaneously capture BOLD and cerebral blood flow (CBF) signals, providing a unique opportunity for assessing resting brain functions with concurrent BOLD (ccBOLD) and CBF signals. Before taking that benefit, it is necessary to validate the utility of ccBOLD signal for resting-state analysis using conventional BOLD (cvBOLD) signal acquired without ASL modulations. To address this technical issue, resting cvBOLD and ASL perfusion MRI were acquired from a large cohort (n = 89) of healthy subjects. Four widely used resting-state brain function analyses were conducted and compared between the two types of BOLD signal, including the posterior cingulate cortex (PCC) seed-based functional connectivity (FC) analysis, independent component analysis (ICA), analysis of amplitude of low frequency fluctuation (ALFF), and analysis of regional homogeneity (ReHo). Consistent default mode network (DMN) as well as other resting-state networks (RSNs) were observed from cvBOLD and ccBOLD using PCC-FC analysis and ICA. ALFF from both modalities were the same for most of brain regions but were different in peripheral regions suffering from the susceptibility gradients induced signal drop. ReHo showed difference in many brain regions, likely reflecting the SNR and resolution differences between the two BOLD modalities. The DMN and auditory networks showed highest CBF values among all RSNs. These results demonstrated the feasibility of ASL perfusion MRI for assessing resting brain functions using its concurrent BOLD in addition to CBF signal, which provides a potentially useful way to maximize the utility of ASL perfusion MRI.

## Introduction

Resting state brain activity represents a major type of brain activity and has attracted enormous research interest in the past decade. Due to its high spatio-temporal resolution and noninvasiveness, BOLD signal based fMRI has become the major tool for assessing resting brain functions. Consistent resting state activity patterns have been repeatedly revealed in different studies [1]–[4], suggesting the existence of an organized mode of resting brain function [5]–[8].

Approximately ten resting-state networks (RSNs) have been reported in independent studies using various hypothesis- and data-driven approaches [4]. Among these networks, the default mode network (DMN) comprising mainly the posterior cingulate cortex/precuneus (PCC/PCu), medial prefrontal cortex (MePFC), and the angular/lateral parietal cortex, has been most reliably reported. Activity in the DMN is proposed to reflect the default brain function in the absence of external stimuli or tasks [9]–[12]. Disturbed DMN function has also been associated with various brain diseases, including Alzheimer's Disease [13]–[15], depression [16]–[18], drug addiction [19]–[22], schizophrenia [23], and stroke [24]. Other reported RSNs include the sensorimotor network, the visual network, the auditory network, the salience network, as well as the attention and executive function networks [4].

ASL fMRI is a non-invasive technique for quantifying cerebral blood flow (CBF) using magnetically labeled arterial blood water as an endogenous tracer. Since CBF is reflective of regional brain function, ASL MRI has been widely used for brain function studies using either the static mean CBF or the dynamic CBF time series [25], [26]. Using resting CBF, our group and others identified similar brain activity patterns to those observed using PET [9], [27], [28], which were further found to be related to several BOLD-imaging derived dynamic resting state measures: including seed-region based FC (SRFC), regional homogeneity (ReHo), and amplitude of low frequency fluctuation (ALFF) [29], suggesting using ASL CBF as a complementary approach for assessing resting brain functions. One noticeable feature of ASL MRI is that the same ASL sequence can acquire both BOLD signal and CBF signal, giving a potential for assessing the dynamic resting brain function (through BOLD) and the static resting state without acquiring additional data. One such acquisition technique is through the dual echo ASL sequence [30], and the other one is to use the concurrent BOLD (ccBOLD) signal acquired in a T2*-weighted gradient echo planar imaging sequence, which is widely used in ASL-based research. While ccBOLD is usually considered a nuisance variable and is removed before CBF quantification, it provides an opportunity for performing data analyses that usually relies on conventional BOLD (cvBOLD) imaging. However, the feasibility and utility of ccBOLD for imaging resting-state brain function has not been quantitatively examined. The purpose of this study was to compare ASL ccBOLD with cvBOLD and validate its utility for resting state brain activity analysis. Standard conventional resting BOLD and ASL data were acquired from the same cohort of normal subjects with similar spatial resolution. Four widely used resting-state brain analyses were performed for quantitative comparisons, including SRFC, ICA, ALFF, and ReHo.

## Materials and Methods

### Subjets

A total of 89 healthy subjects (46 males, mean age  = 28.2, age ranges 20–52 years) participated in this study. All subjects were screened for neurologic and psychiatric conditions. This study was approved by the Institutional Review Board of the University of Pennsylvania. Written informed consent was obtained from each subject before the study.

### MRI data acquisition

MR imaging was conducted in a 3T whole-body scanner (Siemens Medical Systems, Erlangen, Germany). High-resolution structural images were acquired for spatial brain normalization using a 3D MPRAGE sequence. A standard EPI sequence was used for cvBOLD fMRI data acquisition with the following parameters: TR = 0.9 s, TE = 27 ms, FOV = 220×220 mm^2^, matrix  = 64×64×16, slices thickness = 6 mm, inter-slice gap = 1.5 mm. ASL perfusion images were acquired by using a pseudo continuous ASL (pCASL) sequence with the following parameters: TR = 4 s, TE = 17 ms, FOV = 220×220 mm^2^, matrix = 64×64×16, slices thickness  = 6 mm, inter-slice gap  = 1.5 mm, labeling time  = 1.77 s, delay time  = 1.0 s. A total of 360 images and 60 images were acquired in BOLD and pCASL sequence separately. Participants were instructed to lie down still in the scanner at rest and keep eyes open.

### Imaging Data Processing and analyses

Image data processing and analyses were carried out with the Statistical Parametric Mapping software (SPM8, Wellcome Department of Cognitive Neurology, UK) and the REST 2.0 toolbox (http://resting-fmri.sourceforge.net/), implemented in Matlab 14 (Math Works, Natick, MA). The ccBOLD were firstly extracted from ASL data by regressing out the spin labeling paradigm [−1, 1 … −1, 1] from the label/control image series [31]. The cvBOLD and ccBOLD images were then realigned and resliced to correct for head motion. No subject had head motion exceeded 2 mm or rotation exceeded 2.0° during scanning. Structural images were coregistered with the mean volume of functional images and subsequently smoothed using an isotropic Gaussian kernel with a full-width at half-maximum (FWHM) of 4 mm. Images were then normalized to the standard Montreal Neurological Institute (MNI) space and resampled with isotropic 3×3×3 mm^3^ voxel size. Linear trends were also removed. All functional volumes were finally band pass filter at (0.01 Hz < f < 0.08 Hz) to reduce low-frequency drift and physiological high-frequency respiratory and cardiac noise. Nuisance covariates including the six head motion parameters, global mean signal, white matter signal and CSF signal were regressed out from both cvBOLD and ccBOLD [32]. ALFF and ReHo were calculated from the preprocessed data after band pass filtering.

For the ALFF calculation, each voxel's BOLD time series was transformed into the frequency domain and the mean amplitude of the spectrum over the frequency range of 0.01–0.08 Hz was calculated as the ALFF [33].

For the regional coherence calculation, the Kendall's coefficient concordance (KCC, also known as Kendall's W) [34] of each voxel with around 26 nearest neighboring voxels was calculated. The collection of all voxels' Kendall's W formed the so-called ReHo map.

For seed-based FC analysis, the PCC seed was defined as a sphere with a radius of 10 mm located in MNI coordinate (0, −50, 31) [35]. For each individual subject, the mean BOLD fMRI signal time series was extracted from the seed and used as the regressor in the PCC-FC analysis. The correlation coefficients between the time series of PCC and other brain regions were grouped into an individual PCC-FC map, which was transformed into z-score through a Fisher's r-to-z transformation to improve the normality of the correlation coefficients. The same process was repeated for the ccBOLD data. These z-transformed individual FC maps were then entered the second level group analysis using one-sample t-tests. Group-level paired t-test was also conducted to examine the differences of PCC-FC between the cvBOLD and ccBOLD. Threshold was defined as family-wise error (FWE) corrected p<0.05 [36].

For ICA analysis, the preprocessed time series of cvBOLD and ccBOLD after head motion correction, smoothing and spatial normalization were concatenated along time to form a 4-dimensional (4D) dataset. GIFT-toolbox [37] was used to decompose the 4D cvBOLD and ccBOLD data into 20 mutually independent components respectively. All the component maps were transformed to standard z-score and thresholded at z> = 1 for display. These analyses identified 12 RSNs for both cvBOLD and ccBOLD data. CBF maps of each subject were reconstructed from the ASL perfusion data after head motion correction and smoothing with a homebrew toolbox [38], and finally the CBF maps were spatially normalized to MNI space. Mean CBF values of each RSN were extracted for each subject. One-way ANOVA were performed to examine the mean CBF differences across 12 RSNs. Paired t-tests were conducted to compare the CBF differences in each RSN between cvBOLD and ccBOLD.

In order to examine the spatial consistency of the RSNs acquired from cvBOLD and ccBOLD, Dice's similarity coefficient (DSC) was employed [39], [40]. DSC is defined as two times of the intersection volume of the compared RSN maps divided by their sum. The same analysis was performed for both of the group level RSNs and individual level RSNs to evaluate spatial consistency of the networks extracted using ccBOLD and cvBOLD.

## Results

**Figure 1:**
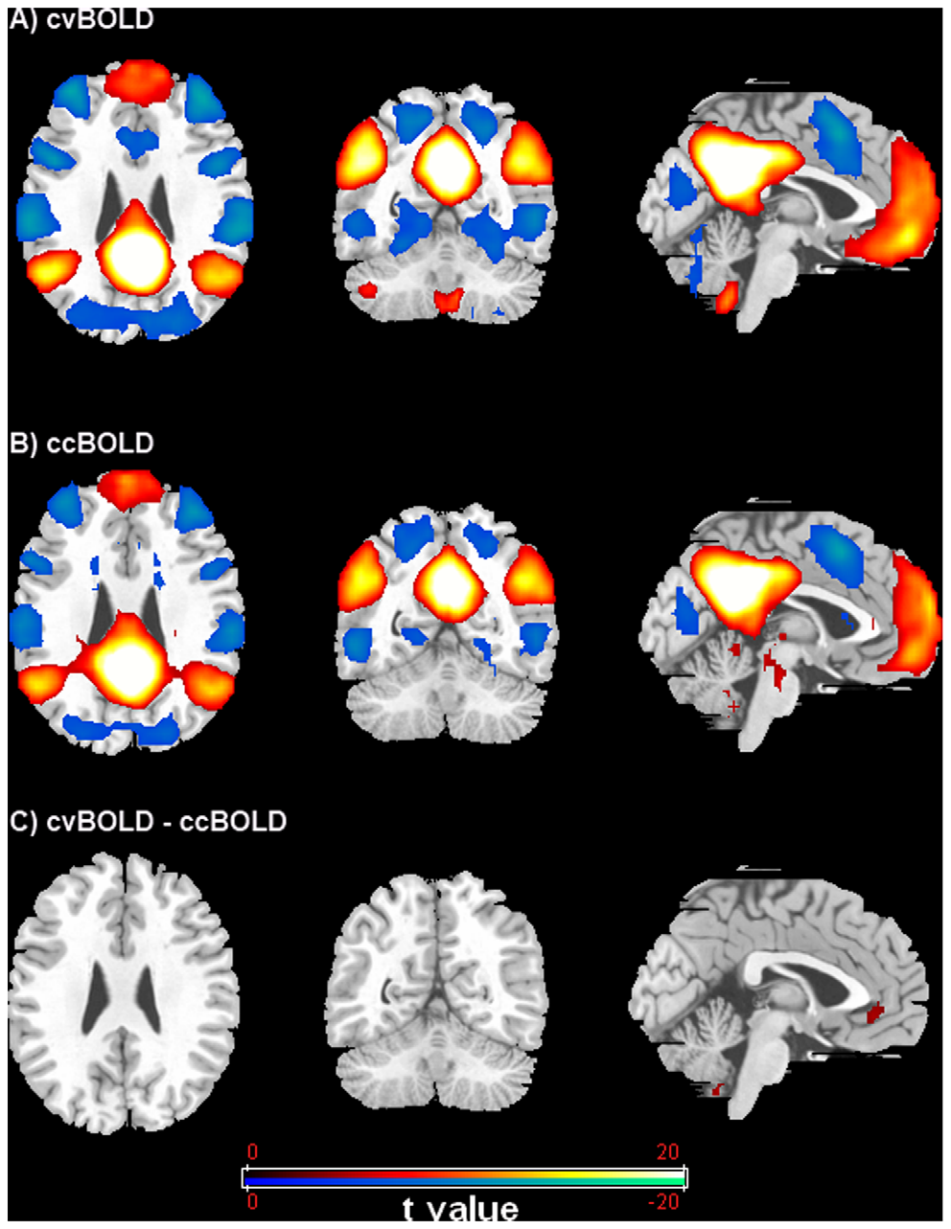
FC results from PCC seed-based analyses showed very similar pattern between conventional BOLD (cvBOLD) (A) and concurrent BOLD (ccBOLD) from ASL data (B). Only minor differences (C) were observed for conventional BOLD comparing with concurrent BOLD (cvBOLD vs. ccBOLD). Threshold was set as FWE corrected p<0.05.

Fig. 1 shows the PCC-FC results for both cvBOLD (Fig. 1A) and ccBOLD (Fig. 1B). The two types of BOLD data produced very similar PCC-FC patterns, which is generally dubbed as the DMN, with minor disparities. The Dice index between the PCC-FC of the cvBOLD and ccBOLD was 0.77. A direct comparison of the two modalities regarding the PCC-FC patterns showed a few scattered brain regions with higher PCC-FC in cvBOLD than ccBOLD, including the left medial orbital frontal cortex, right caudate and left cerebellum area (Fig.1C and Table 1).

**Table 1:** Peak MNI coordinates of the 4 clusters of the paired t-test result of cvBOLD and ccBOLD (p = 0.05, k = 15, FWE-corrected).

Regions	Cluster size	Peak t	MNI Coordinates		
			x	y	z
Left Medial Orbital Frontal	33	5.78	0	36	−9
Right Caudate	17	4.71	12	18	9
Left Cerebellum	31	5.73	−6	−54	−54

**Figure 2:**
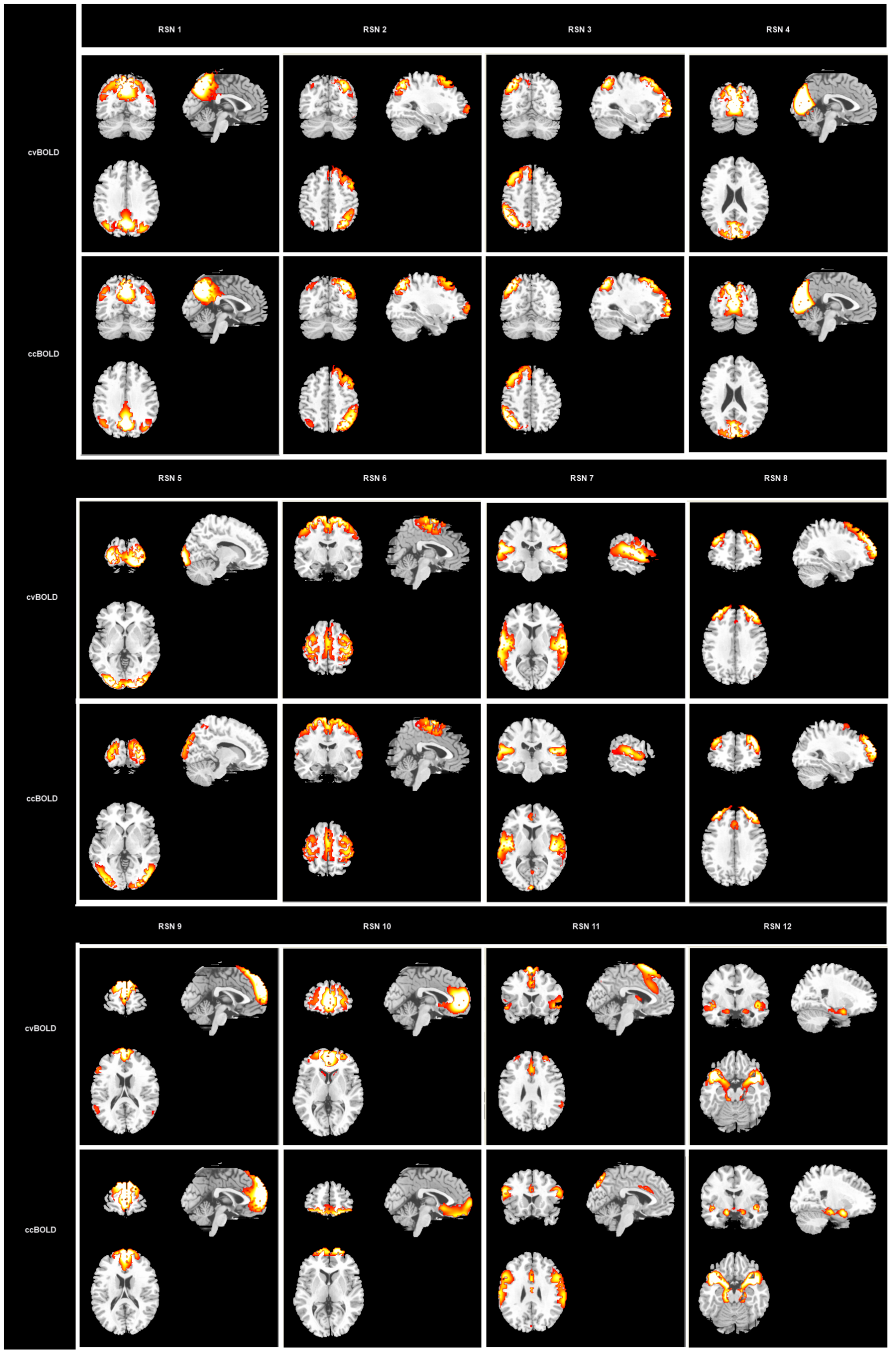
Resting-state networks (RSNs) identified from ICA analysis of conventional BOLD (up-levels) and concurrent BOLD (lower-levels) data. RSN1: default mode network (DMN); RSN 2: left attention networks; RSN 3: right attention network; RSN 4: primary visual network; RSN 5: secondary visual network; RSN 6: sensorimotor network; RSN 7: auditory network; RSN 8: executive network; RSN 9: dorsal medial prefrontal network (DMPFC); RSN 10: ventral medial prefrontal network (VMPFC); RSN 11: salience network, RSN 12: medial temporal limbic network.

Fig. 2 shows the ICA-derived group level RSNs using both types of BOLD signals. After visually excluding the noise components based on the literature [41], 12 RSNs were identified for both cvBOLD and ccBOLD, including the DMN (RSN 1), left and right attention networks (RSN 2 and 3), primary and secondary visual networks (RSN 4 and 5), sensorimotor network (RSN 6), auditory network (RSN 7), executive network (RSN 8), dorsal and ventral medial prefrontal network (DMPFC and VMPFC, RSN 9 and 10), salience network (RSN 11), and the medial temporal limbic network (RSN 12).

**Figure 3:**
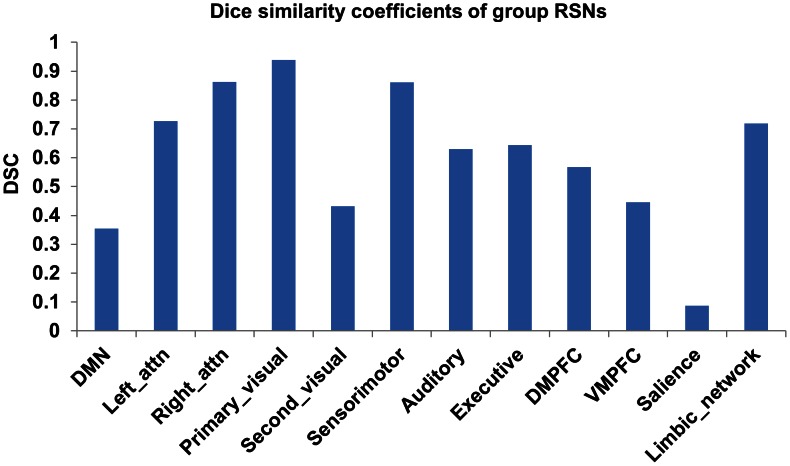
The Dice's similarity coefficients of the 12 RSNs between two BOLD modalities at the group level.

**Figure 4:**
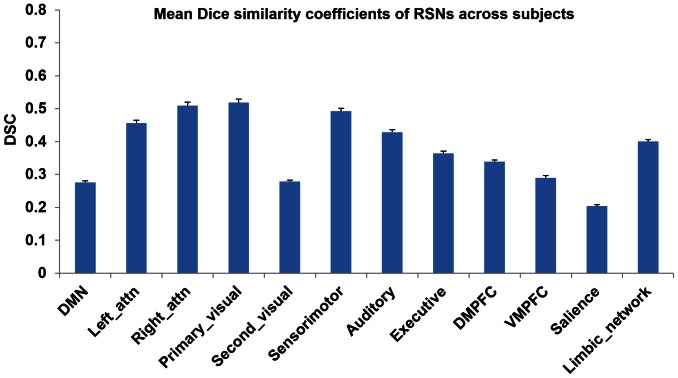
The averaged Dice's similarity coefficients of the 12 RSNs between two BOLD modalities at the individual level.

Fig. 3 shows the Dice index for the group level RSNs. Consistent with Fig. 2, the RSNs derived from both types of data were highly stable. Most of the RSNs had good spatial overlap consistency (DSC>0.3) except the salience network (DSC = 0.09). Similar across-modality reproducibility of these RSNs from each individual subjects was observed in the mean and standard error of the Dice index as shown in Fig. 4.

**Figure 5:**
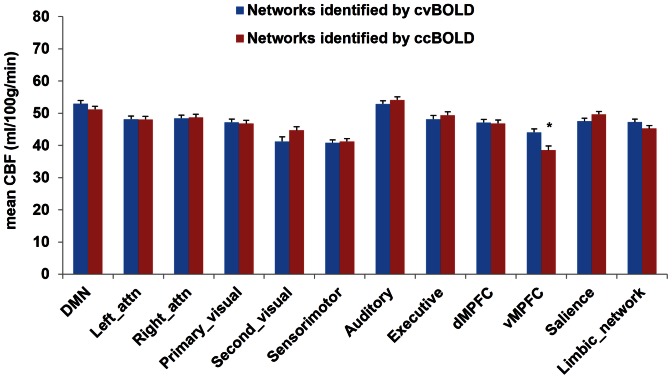
Mean CBF values extracted from the 12 resting-state networks by ICA analyses. Significant difference was observed only in the VMPFC, with concurrent BOLD (ccBOLD) showed lower CBF values than conventional BOLD (cvBOLD). Error bar represented standard error. * p<0.05.

The mean CBFs extracted from the 12 RSNs of both cvBOLD and ccBOLD were illustrated in Fig. 5. No significant CBF difference was observed except in the VMPFC network (p<0.05 after multiple-comparison correction). One-way ANOVA analysis showed that mean CBF values were significantly different across the 12 RSNs for both of the cvBOLD and ccBOLD (both p<0.0001). Among these RSNs, DMN and auditory networks showed the highest CBF while the sensorimotor network, secondary visual network, and VMPFC were the lowest ones.

**Figure 6:**
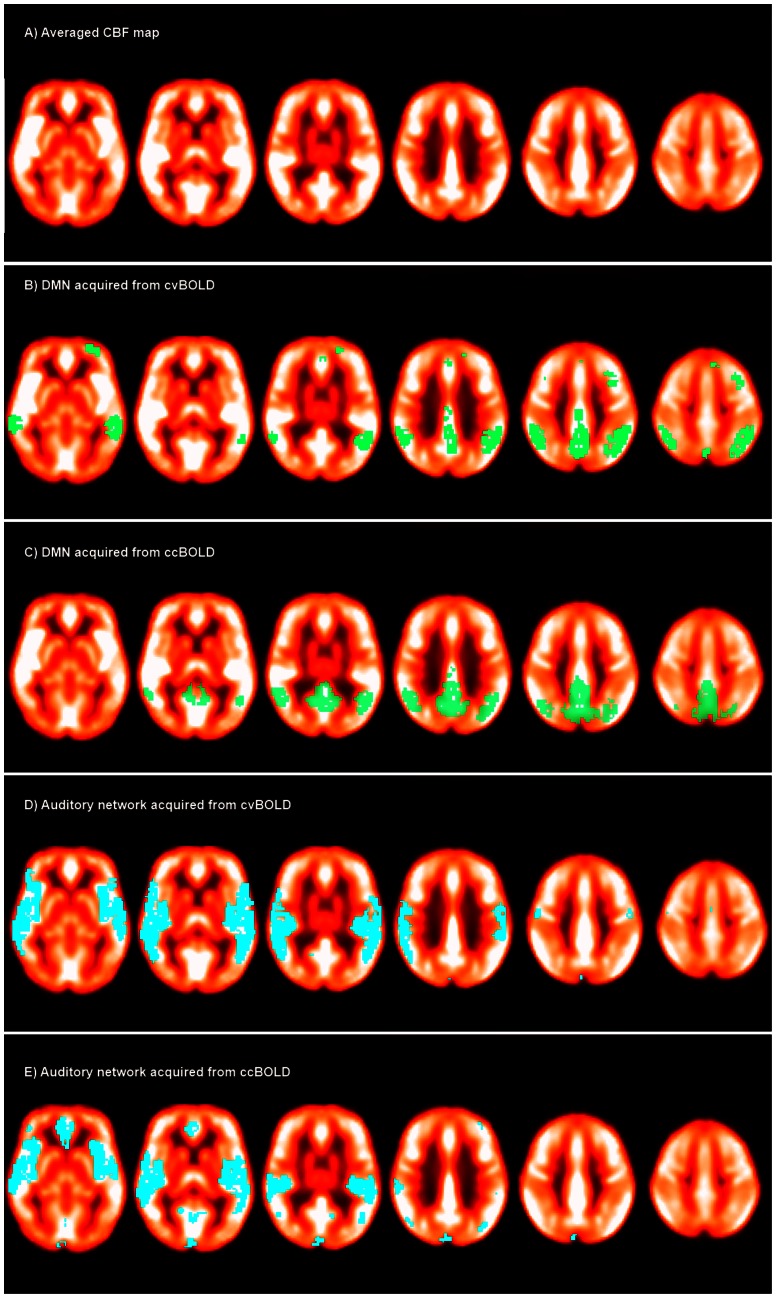
The map of averaged CBF across subjects (A) and the overlays of DMN (B & C) and auditory network acquired from cvBOLD and ccBOLD (D & E).

To directly show the CBF distributions across different RSNs, DMN and auditory network, which had the highest CBF as compared to other RSNs, were overlaid on the mean CBF map of all subjects in Fig.6.

**Figure 7:**
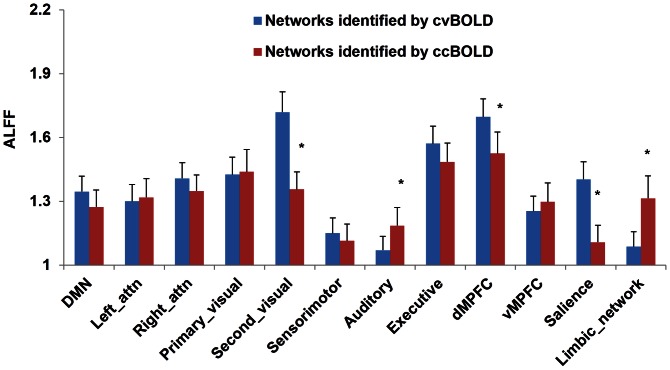
Mean ALFF values extracted from the 12 resting-state networks by ICA analyses. Significant difference was observed in the primary visual, auditory, DMPFC, salience and limbic network. Error bar represented standard error. * p<0.05.

**Figure 8:**
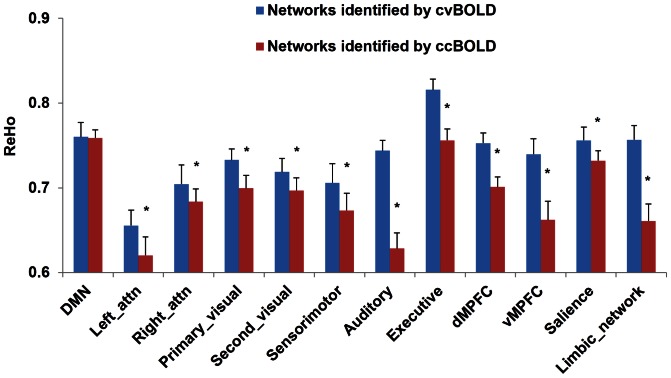
Mean ReHo values extracted from the 12 resting-state networks by ICA analyses. Significant difference was observed in all RSNs except DMN network. Error bar represented standard error. * p<0.05.

ALFF and ReHo analysis yielded different results using cvBOLD and ccBOLD. Fig. 7 and 8 show the mean ALFF and ReHo within the 12 ICA-derived RSNs, respectively. Significant ALFF difference was observed in the primary visual, auditory, DMPFC, salience and limbic network (p<0.05 after multiple-comparison correction), and significant ReHo difference was observed in all RSNs (p<0.05 after multiple-comparison correction) except in the DMN network.

## Discussion

Using data from a large cohort of 89 subjects, we demonstrated that the concurrent BOLD signal from the T2*-weighted 2D EPI-based ASL MRI can reliably detect DMN and other frequently reported RSNs as compared to conventional BOLD. These results suggest an effective way to assess resting brain FC and RSNs using the T2* weighted ASL MRI sequence, which is very useful for studies with limited scan time where only ASL data can be acquired.

PCC-FC has been shown to be a very stable resting state measures [3], [11], [28], [42]–[44], and was selected for the resting FC analysis in this study. The group level PCC-FC results from both types of data were very similar with some minor differences in FC strengths. These differences might be due to the SNR and degree- of-freedom differences between these two types of BOLD data. The ASL MRI acquisition used a shorter TE than the optimal TE used in the conventional BOLD imaging acquisition, resulting in a relatively weaker BOLD signal in ccBOLD. Meanwhile, ccBOLD had much lower temporal resolution and 6 times fewer time-points than conventional BOLD. The cross-modality resting state brain activity analysis comparison suggests that PCC-FC is a stable resting state brain activity analysis method that can be examined with data with different SNR (determined by the acquisition echo time) and different temporal resolution even with only 60 time-points.

ICA based RSN analysis is stable across modalities as well. However, these findings do not suggest using ICA for cross-population RSN comparisons since our previous test-retest study[42] showed that ICA-derived RSNs had a poor voxel-wise test-retest stability, which might be partly caused by the scale ambiguity of ICA. The stable across-modality ICA RSN patterns rather suggest using them as ROIs for assessing changes of other physiological measures with those networks. One such example was shown in the RSN-based CBF analysis. Consistent with previous findings [43], [44], DMN showed higher CBF than other RSNs except for the auditory network, supporting that DMN is the most prominent active network during the resting state. High CBF in auditory networks has also been reported in other resting brain imaging studies [45], which may be due to the unavoidable noise during MR scanning. Nevertheless, the systematic CBF differences observed between these RSNs suggest that RSN-based CBF may be a potential marker for regional brain activity changes in cross-sectional or longitudinal studies.

ALFF and ReHo were mostly not comparable across cvBOLD and ccBOLD. One potential reason for the ALFF inconsistency is differences in the temporal resolution and sampling rate. cvBOLD had much higher temporal resolution than ccBOLD, which consequently captured wider power spectrum of the underlying resting brain activity. Since ALFF is directly derived from the power spectrum, any substantially alterations to the acquisition frequency band would bring significant changes to ALFF value. The different number of BOLD images directly affects the temporal SNR, which affects the power spectrum estimation too. ReHo depends on the data coherences within a prior regional neighborhood and it can be expected to decrease when SNR drops or more time points are included (like in cvBOLD as compared to ccBOLD). Since noise is unavoidable in fMRI, more time points means more discrepancies to the regional data coherence, which inevitably induces a globally ReHo value drop. Although we can intentionally match the number of time points for both cvBOLD and ccBOLD, the sampling rate and SNR differences would still bring a systematic difference between ALFF and ReHo values of the two BOLD modalities as the two measures.

Our results of using the fewer temporal points to reliably assess RSNs (using PCC-FC or ICA) are consistent with Tagliazucchi et. al (2012) [46], where evidence was shown for reliably revealing resting brain activity patterns using a small portion of data. By performing ICA at the group level (using the concatenated data) and the individual level (using each subject's data separately), we showed high reproducibility of the RSNs measured with the Dice index. A DSC>0.3 is generally considered as good overlap [47]. Our results showed that both the group level RSNs and individual level RSNs had DSC>0.3. The individual level cvBOLD vs ccBOLD RSN Dice index also showed a very small variations, suggesting a stable assessment of those RSNs using both types of BOLD signal at the individual level.

In summary, this study demonstrates the utility of concurrent BOLD and CBF signals from ASL perfusion MRI for assessing resting brain function. Although not studied in this work, the CBF time series may be also used for resting brain activity analysis. As BOLD is affected by macro-vascular effects, CBF reflects activity more in tissue capillary bed. Therefore ASL CBF may provide a more sensitive approach to measure differences in resting brain activity or RSNs, although this need be assessed in future works.
